# Associations among body composition parameters and quality of life in peritoneal dialysis patients

**DOI:** 10.1038/s41598-022-19715-2

**Published:** 2022-11-10

**Authors:** Seon-Mi Kim, Minjung Kang, Eunjeong Kang, Ji Hye Kim, Yunmi Kim, Hyunjin Ryu, Seung Seok Han, Hajeong Lee, Yong Chul Kim, Kook-Hwan Oh

**Affiliations:** 1 Department of Internal Medicine, Seoul National University Hospital, 101 Daehakro, Jongno-Gu, Seoul, 03080 Republic of Korea; 2 Department of Internal Medicine, Ewha Womans University Seoul Hospital, Seoul, Republic of Korea; 3 Department of Internal Medicine, Inje University Busan Paik Hospital, Busan, Republic of Korea

**Keywords:** Kidney diseases, Renal replacement therapy

## Abstract

Health-related quality of life (HRQOL) is an important issue among patients undergoing dialysis treatment. Peritoneal dialysis (PD) is associated with a number of adverse body composition changes. However, whether body composition is associated with HRQOL is uncertain. The purpose of this study was to analyze the effects of body composition on HRQOL in PD patients. We performed a cross-sectional observational study on the association between body composition and HRQOL in PD patients at a single center. Body composition was determined by multifrequency bioimpedance spectroscopy. HRQOL is summarized to three composite scores: kidney disease component summary (KDCS), physical component summary (PCS), and mental component summary (MCS). The relationships between HRQOL and the hydration index, lean tissue index (LTI), and fat tissue index (FTI) were analyzed by regression analysis. One hundred and ninety-seven PD patients were included in the present study. Patients with severe fluid overload showed a lower PCS. The hydration index and FTI showed statistically significant negative associations with PCS. In subgroup analysis, the associations between the hydration index and PCS remained robust after stratifying according to sex, age, and residual urine. Our results indicated that both the hydration index and FTI were negatively associated with HRQOL, especially PCS.

## Introduction

The concept of health-related quality of life (HRQOL) addresses the effects of individual health on physical, cognitive, and social functioning in daily life^1^. Patients with end-stage kidney disease (ESKD) who are treated with dialysis have many threats to HRQOL, both from the ESKD-related symptoms per se and from the physical and mental burden of dialysis treatment^1,2^. The importance of HRQOL to kidney disease patients is obviously not only the basic aspect of the goal itself, but also the association with morbidity and mortality^3–6^. The International Society for Peritoneal Dialysis (ISPD) has also recently published practice recommendations on HRQOL to patients undergoing peritoneal dialysis (PD)^7^. The ISPD recommended that high-quality PD prescriptions should be guided by a number of assessments encompassing the person’s volume and nutritional status.

PD patients encounter complex body composition changes, with overhydration, muscle wasting, and fat gain^8–10^. The assessment of body composition is essential to effectively evaluate nutritional status in PD patients and monitor changes during dialysis^11^. Body mass index (BMI) is one of the most popular methods for evaluating nutrition status. However, BMI is not able to differentiate between volume overload and obesity and is less associated with muscle wasting in dialysis patients^12,13^. Therefore, access to specific indicators of body composition to evaluate volume and nutritional status is needed. Multifrequency bioimpedance spectroscopy (BIS) with a body composition model is a validated method to assess hydration and nutritional status in dialysis patients^14,15^. It has anthropometric parameters to assess both fluid volume, such as tissue hydration, extracellular water (ECW), and intracellular water (ICW), and nutritional status such as lean tissue inde× (LTI) and fat tissue index (FTI).

Whether body composition is associated with HRQOL in PD patients remains unclear. Therefore, the aim of the current study was to evaluate whether body fluid status or nutritional indices assessed by BIS may affect HRQOL in PD patients. To do so, we analyzed the data from patients who are enrolled in the Korean Cohort Study for Outcome in Patients on Peritoneal Dialysis (KNOW-PD) cohort.

## Results

### Baseline profile of the study population

We screened subjects registered in the KNOW-PD cohort, and 197 PD patients passed the inclusion and exclusion criteria and were involved in this study. Table 1 shows the baseline characteristics, laboratory findings, and BIS measurements of the patients. The study population included 54.8% males, and the mean age was 45.8 ± 13.5 years. The median dialysis vintage was 31 months. The assessment of nutritional status revealed 92.4% of subjects having SGA 6–7, which indicates a well-nourished status. 69.5% of subjects were treated with renin-angiotensin aldosterone systems (RAAS) blockade with angiotensin-converting enzyme inhibitors and/or angiotensin II receptor blockers. 79.2% of subjects were treated with erythropoiesis stimulating agents. According to the BIS parameters, the mean R∆TH of patients was 13.4 ± 10.2%, and the median A∆TH was 2.3 (0.9, 3.5) L, which indicates a fluid overload status. Mean FTI was 7.7 ± 3.8 kg/m^2^, and the Z-FTI (Z-score of fat tissue index) was − 0.2 ± 1.1. Mean LTI was 14.3 ± 3.0 kg/m^2^, and the Z-LTI (Z-score of lean tissue index) was − 0.5 ± 1.2.

**Table 1:** Baseline demographic and biochemical details of the study subjects.

Characteristics	*N* = *197*
Male	108 (54.8%)
Age (years)	45.8 ± 13.5
Height (cm)	164.0 ± 8.7
Weight (kg)	62.0 ± 12.9
**Blood pressure**	
Systolic (mmHg)	139.6 ± 19.8
Diastolic (mmHg)	83.9 ± 13.4
Dialysis duration (month)	31 (1, 63)
**Peritoneal dialysis modality**	
Continuous ambulatory peritoneal dialysis	92 (46.7%)
Continuous cycling peritoneal dialysis	42 (21.3%)
Intermittent peritoneal dialysis	53 (26.9%)
Night intermittent peritoneal dialysis	10 (5.1%)
**Kt/V**	
Peritoneal	1.5 (1.0, 1.7)
Renal	0.5 (0.0, 1.1)
Total	2.0 (1.7, 2.4)
**Creatinine clearance (mL/min/1.73 m**^**2**^**)**	
Peritoneal	40.3 (25.4, 48.0)
Renal	24.9 (0.3, 61.7)
Total	55.2 (46.3, 83.7)
24-h urine volume (mL/day)	750 (0.1, 200)
Glucose exposure load (g/day)	89.4 (60.1, 114.1)
History of icodextrin bag usage	100 (50.8%)
**Type of membrane transport**	
High	57 (28.9%)
High average	66 (33.5%)
Low average	42 (21.3%)
Low	14 (7.1%)
Missing	18 (9.1%)
**Subjective global assessment**	
4–5	8 (4.1%)
6	41 (20.8%)
7	141 (71.6%)
Missing	7 (3.6%)
Normalized protein nitrogen appearance(g/kg/day) (*n* = 167)	0.99 ± 0.2
Diabetes mellitus	50 (25.4%)
**Davies score**	
1	98 (49.7%)
2–3	96 (48.7%)
≥ 4	3 (1.5%)
**Laboratory finding**	
Albumin (g/dL)	3.7 ± 0.4
BUN (mg/dL)	57.8 ± 16.2
C-reactive protein (mg/dL)	0.1(0.0, 0.2)
Hemoglobin (g/dL)	10.8 ± 1.5
Sodium (mmol/L)	138.0 (134.0, 140.0)
Potassium (mmol/L)	1.5 ± 0.7
Cholesterol (mg/dL)	164.8 ± 41.9
Triglyceride (mg/dL) (*n* = 160)	112.5 (76.3, 161.0)
Low density lipoprotein (mg/dL) (*n* = 155)	89.2 ± 33.0
High density lipoprotein (mg/dL) (*n* = 159)	47.0 (39.0, 57.0)
Left ventricular mass index (g/m^2^) (*n* = 165)	107.1 (84.3. 130.1)
**Antihypertensive**	
On angiotensin receptor blocker	131 (66.5%)
On angiotensin-converting enzyme inhibitor	6 (3.0%)
On ß-blocker	49 (24.9%)
On calcium channel blocker	137 (69.5%)
On diuretics	10 (5.1%)
Erythropoiesis stimulating agents	156 (79.2%)
**Bioimpedance measurements**	
Total body water (L)	34.2 ± 7.7
Extracellular water (L)	16.3 ± 4.0
Intracellular water (L)	17.5 (15.2, 21.2)
Extracellular water/intracellular water	0.9 ± 0.2
Relative ∆tissue hydration (R∆TH, %)	13.4 ± 10.2
Absolute ∆tissue hydration (A∆TH, L)	2.3 (0.9,3.5)
Fat tissue index (kg/m^2^)	7.7 ± 3.8
Z-score of fat tissue index	− 0.2 ± 1.1
Lean tissue index (kg/m^2^)	14.3 ± 3.0
Z-score of lean tissue index	− 0.5 ± 1.2

Continuous variables are expressed as mean ± standard deviations (normal distribution) or as median and interquartile (non-normal distribution). Categorical variables are expressed as number, (%).

### HRQOL scores according to body composition

Table 2 shows the comparison of KDQOL-SF classified according to R∆TH, ECW/ICW, Z-FTI, and Z-LTI. KDCS and MCS showed no significant differences in the groups classified by R∆TH, ECW/ICW, Z-FTI, and Z-LTI. However, PCS varied significantly between the group with R∆TH ≤ 15% and the group with R∆TH > 15%, as well as between the group with ECW/ICW < 1 and the group with ECW/ICW ≥ 1. The subscales of the KDQOL-SF classified according to R∆TH, ECW/ICW, Z-FTI, and Z-LTI were also compared (Supplement Table S1). The R∆TH > 15% group showed statistically significantly lower scores in two PCS components, physical functioning and general health. The subjects with ECW/ICW ≥ 1 also showed statistically significant lower scores in two PCS components, physical functioning and role-physical.

**Table 2:** HRQOL scores: Comparison between various body composition status.

	Relative ∆tissue hydration			ECW/ICW			Z-FTI			Z-LTI		
	R∆TH ≤ 15% (n = 118)	R∆TH > 15% (n = 79)	*p*	ECW/ICW < 1 (n = 144)	ECW/ICW ≥ 1 (n = 53)	*P*	Z-FTI < 0 (n = 121)	Z-FTI ≥ 0 (n = 76)	*p*	Z-LTI < 0 (n = 128)	Z-LTI ≥ 0 (n = 69)	*p*
KDCS	718.3 ± 131.5	688.6 ± 134.0	0.125	706.8 ± 130.7	705.5 ± 140.2	0.952	705.5 ± 133.9	707.9 ± 132.4	0.903	709.4 ± 135.4	701.0 ± 129.1	0.673
PCS	227.5 (174.8,305.0)	195.0 (160.0, 252.5)	0.012	227.5 (176.0,298.8)	203.2 ± 79.7	0.01	211.3 (162.5, 260.0)	214.5 ± 71.7	0.077	220.0 (175.6,287.5)	212.5 (163.8,277.5)	0.358
MCS	248.3 (167.4,305.0)	220.2 (142.5,277.0)	0.058	243.3 (156.3,286.9)	218.0 (140.5,310.5)	0.7	252.5 (163.7,292.8)	207.9 (136.1,292.6)	0.109	243.3 (148.1,296.9)	220.2 (156.0,289.8)	0.746

*HRQOL* health related quality of life, *R∆TH* Relative ∆tissue hydration, *ECW* extracellular water, *ICW* intracellular water, *Z-FTI* Z-score of fat tissue index, *Z-LTI* Z-score of lean tissue index, *KDCS* kidney disease component summary, *PCS* physical component summary, *MCS* mental component summary.

### Correlations between body composition and KDQOL scores

The correlation coefficients between the body composition parameters and each component of KDQOL-SF are shown in Table 3. In brief, we found that KDCS had no correlation with any of the body composition parameters. PCS showed a negative correlation with R∆TH (Spearman’s r = − 0.3, *p* < 0.001), ECW/ICW (Spearman’s r = − 0.3, *p* < 0.001), and Z-FTI (Spearman’s r = − 0.2, *p* = 0.005). MCS was weakly associated with R∆TH (Spearman’s r = − 0.2, *p* = 0.016), ECW/ICW (Spearman’s r = − 0.2, *p* = 0.034), and Z-FTI (Spearman’s r = − 0.2, *p* = 0.036).

**Table 3:** Correlation of body composition parameters with HRQOL components.

	Relative ∆tissue hydration		ECW/ICW		Z-FTI		Z-LTI	
	Spearman’s r	*p*	Spearman’s r	*p*	Spearman’s r	*p*	Spearman’s r	*p*
KDCS	− 0.1	0.061	− 0.1	0.172	− 0.1	0.417	− 0.1	0.408
PCS	− 0.3	< 0.001	− 0.3	< 0.001	− 0.2	0.005	0.1	0.624
MCS	− 0.2	0.016	− 0.2	0.034	− 0.2	0.036	0.1	0.366

*HRQOL* health related quality of life, *ECW* extracellular water, *ICW* intracellular water, *Z-FTI* Z-score of fat tissue index, *Z-LTI* Z-score of lean tissue index, *CI* confidence interval, *KDCS* kidney disease component summary, *PCS* physical component summary, *MCS* mental component summary.

To evaluate whether body composition was associated with HRQOL scores, linear regression analysis was performed (Table 4). We adjusted several factors which could affect the HRQOL in several models. Activation of the RAAS in particular increased angiotensin II levels and stimulated growth of myocardial cells, which caused the left ventricular hypertrophy^16^. Blockade of RAAS in PD patients are likely to preserve residual renal function and reduce left ventricular mass^17–19^, which could affect the body composition of PD patients. Therefore, we performed a linear regression analysis by adding the usage of RAAS blockade as an adjustment variable to model 5. After adjustments for age, sex, dialysis duration, Kt/V, total creatinine clearance, C-reactive protein, body composition parameters, diabetes mellitus, and usage of RAAS blockade (Model 5), R∆TH and ECW/ICW, expressed in the hydration index, showed statistically significant negative associations with PCS, with β = − 1.5 (− 2.8, − 0.1) and β = − 117.2 (− 215.2, − 19.2), respectively. In Model 5, Z-FTI showed a statistically significant negative association with PCS, with β = − 14.3 (− 28.3, − 0.3). FTI showed a similar trend as Z-FTI with PCS (data not shown). Neither LTI (data not shown) nor Z-LTI had a statistically significant association with any component of HRQOL.

**Table 4:** Regression coefficients of the body composition parameters for HRQOL scores.

Predictor variable	Relative ∆tissue hydration		ECW/ICW		Z-FTI		Z-LTI	
	β (95% CI)	*p*	β (95% CI)	*p*	β (95% CI)	*p*	β (95% CI)	*p*
**KDCS**								
Model 1	− 1.4 (− 3.5, 0.7)	0.184	− 55.5 (− 197.7, 86.6)	0.442	− 2.8 (− 22.3, 16.6)	0.773	− 5.1 (− 23.0, 12.8)	0.572
Model 2	− 1.5 (− 3.6, 0.6)	0.161	− 57.3 (− 205.4, 90.8)	0.446	− 2.6 (− 22.0, 16.8)	0.790	− 6.0 (− 24.0, 12.0)	0.510
Model 3	− 1.6 (− 1.0, 2.2)	0.15	− 64.3 (− 215.4, 86.8)	0.402	− 4.5 (− 24.8, 15.7)	0.659	− 5.2 (− 23.4, 13.1)	0.575
Model 4	− 1.6 (− 3.9, 0.5)	0.159	− 75.6 (− 248.4, 97.1)	0.388	− 8.4 (− 30.7, 13.9)	0.459	− 10.7 (− 30.9, 9.6)	0.301
Model 5	− 0.8 (− 3.2, 1.6)	0.495	6.5 (− 167.6, 180.6)	0.941	− 1.2 (− 22.5, 20.0)	0.909	− 7.6 (− 26.0, 10.7)	0.413
**PCS**								
Model 1	− 2.0 (− 3.1, − 0.8)	0.001	− 141.9 (− 220.8, − 62.9)	0.001	− 13.1 (− 24.1, − 2.0)	0.021	0.1 (− 10.4, 10.3)	0.989
Model 2	− 2.0 (− 3.1, − 0.8)	0.001	− 148.9 (− 231.8, − 66.0)	0.001	− 13.1 (− 24.2, − 2.0)	0.021	0.2 (− 10.3, 10.6)	0.976
Model 3	− 2.0 (− 3.1, − 0.7)	0.002	− 146.8 (− 231.5, − 62.1)	0.001	− 12.7 (− 24.3, − 1.0)	0.033	0.2 (− 10.4, 10.8)	0.970
Model 4	− 2.0 (− 3.2, − 0.7)	0.002	− 142.9 (− 239.2, − 46.7)	0.004	− 15.2 (− 27.6, − 2.7)	0.017	− 8.2 (− 19.5, 3.1)	0.154
Model 5	− 1.5 (− 2.8, − 0.1)	0.036	− 117.2 (− 215.2, − 19.2)	0.019	− 14.3 (− 28.3, − 0.3)	0.048	− 6.0 (− 24.0, 12.0)	0.510
**MCS**								
Model 1	− 1.2 (− 2.5, 0.1)	0.068	− 77.6 (− 165.4, 10.1)	0.083	− 8.1 (− 20.2, 3.9)	0.183	− 3.5 (− 14.6, 7.7)	0.538
Model 2	− 1.2 (− 2.5, 0.1)	0.068	− 85.1 (− 177.2, 7.0)	0.070	− 8.1 (− 20.3, 4.0)	0.187	− 3.6 (− 14.9, 7.7)	0.534
Model 3	− 1.2 (− 2.5, 0.1)	0.076	− 84.8 (− 178.7, 9.1)	0.076	− 8.0 (− 20.7, 4.7)	0.213	− 3.5 (− 14.9, 7.9)	0.547
Model 4	− 1.3 (− 2.6, 0.05)	0.059	− 86.4 (− 193.1, 20.4)	0.112	− 11.7 (− 25.5, 2.2)	0.098	− 9.7 (− 22.2, 2.9)	0.13
Model 5	− 0.9 (− 2.4, 0.6)	0.249	− 61.6 (− 170.4, 47.2)	0.265	− 5.3 (− 18.6, 8.0)	0.43	− 9.5 (− 22.4, 3.4)	0.148

Model 1: Unadjusted.

Model 2: Adjusted for age and sex.

Model 3: Adjusted for Model 2 plus dialysis duration, Kt/V, total creatinine clearance, and C-reactive protein.

Model 4: Adjusted for Model 3 plus relative ∆tissue hydration, Z-FTI, and Z-LTI.

Model 5: Adjusted for Model 4 plus diabetes mellitus and usage of renin–angiotensin–aldosterone system blockade.

*HRQOL* health related quality of life, *ECW* extracellular water, *ICW* intracellular water, *Z-FTI* Z-score of fat tissue index, *Z-LTI* Z-score of lean tissue index, *CI* confidence interval, *KDCS* kidney disease component summary, *PCS* physical component summary, *MCS* mental component summary.

### Stratified subgroup analysis

**Figure 1:**
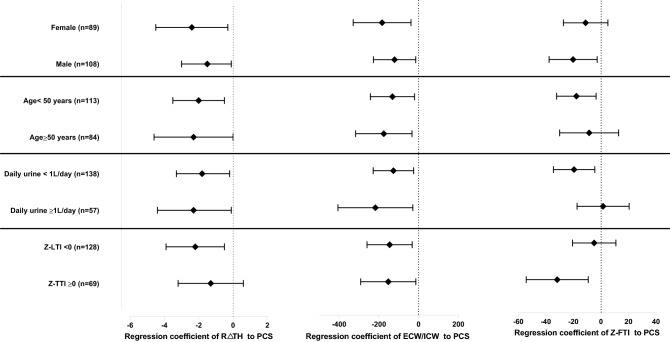
Plot of regression coefficients for the association between PCS and R∆TH, ECW/ICW, and Z-FTI. Error bars indicate 95% confidence intervals. *R∆TH* Relative ∆tissue hydration, *ECW* extracellular water, *ICW* intracellular water, *Z-FTI* Z-score of fat tissue index, *Z-LTI* Z-score of lean tissue index, *PCS* physical component summary.

After stratifying the subjects according to sex, age, daily urine, and Z-LTI, we performed a subgroup analysis of linear regression to determine the association between the status of fluid volume/nutrition and HRQOL (Table 5). Associations between R∆TH and PCS remained robust after stratifying according to sex, age, and residual urine (Fig. 1). The associations between ECW/ICW and PCS also remained robust after stratifying according to age, residual urine, and Z-LTI. Z-FTI and PCS showed negative associations in the following subgroups: males, age under 50 years, residual daily urine < 1 L/day, and Z-LTI ≥ 0.

**Table 5:** Subgroup analysis of regression coefficient of the body composition parameters for HRQOL scores.

Stratification	Predictor variable	Relative ∆tissue hydration		ECW/ICW		Z-FTI	
		β (95% CI)	*p*	β (95% CI)	*p*	β (95% CI)	*p*
**Sex**							
Female (n = 89)	KDCS	− 1.6 (− 5.2, 2.0)	0.373	− 72.0 (− 325.6, 181.5)	0.572	1.6 (− 25.7, 28.8)	0.908
	PCS	− 2.4 (− 4.5, − 0.3)	0.003	− 183.7 (− 329.2, − 38.1)	0.014	− 11.1 (− 27.2, 5.1)	0.176
	MCS	− 1.3 (− 3.5, 1.0)	0.26	− 108.7 (− 263.4, 46.0)	0.165	− 9.8 (− 26.4, 6.9)	0.247
Male (n = 108)	KDCS	− 1.1 (− 3.7, 1.5)	0.409	− 54.9 (− 240.5, 130.7)	0.558	− 18.3 (− 48.6, 12.0)	0.233
	PCS	− 1.5 (− 3.0, − 0.1)	0.045	− 120.8 (− 227.3, − 14.3)	0.027	− 20.2 (− 37.6, − 2.7)	0.025
	MCS	− 1.0 (− 2.3, 0.9)	0.371	− 57.6 (− 172.4, 57.2)	0.321	− 12.0 (− 30.8, 6.8)	0.207
**Age**							
Age < 50 (n = 113)	KDCS	− 1.0 (− 3.8, 1.9)	0.507	− 19.0 (− 235.4, 197.3)	0.862	− 17.7 (− 45.4, 9.9)	0.206
	PCS	− 2.0 (− 3.5, − 0.5)	0.008	− 131.5 (− 242.4, − 20.6)	0.021	− 3.3 (− 24.0, 17.4)	0.749
	MCS	− 0.9 (− 2.5, 0.7)	0.279	− 34.3 (− 158.6, 90.0)	0.585	− 13.9 (− 29.7, 1.9)	0.084
Age ≥ 50 (n = 84)	KDCS	− 3.4 (− 7.0, 0.2)	0.062	− 136.5 (− 364.6, 91.7)	0.236	14.3 (− 18.6, 47.3)	0.387
	PCS	− 2.3 (− 4.6, − 0.0)	0.049	− 175.0 (− 316.8, − 33.3)	0.017	− 4.4 (− 30.3, 21.5)	0.734
	MCS	− 2.1 (− 4.5, 0.2)	0.078	− 153.7 (− 300.5, − 6.9)	0.04	− 4.2 (− 26.1, 17.7)	0.7
**Residual renal function**							
Daily urine < 1 L/day (n = 138)	KDCS	− 1.1 (− 3.9, 1.7)	0.426	− 30.6 (− 214.7, 153.5)	0.742	− 11.1 (− 38.3, 16.1)	0.419
	PCS	− 1.8 (− 3.3, − 0.2)	0.027	− 126.8 (− 228.6, − 25.0)	0.015	− 19.0 (− 36.5, − 1.4)	0.035
	MCS	− 1.1 (− 2.8, 0.6)	0.185	− 75.6 (− 186.8, 35.5)	0.18	− 14.1 (− 30.5, 2.3)	0.091
Daily urine ≥ 1 L/dayay (n = 57)	KDCS	− 2.9 (− 6.5, 0.8)	0.119	− 216.5 (− 540.0, 106.9)	0.184	7.3 (− 24.0, 38.5)	0.642
	PCS	− 2.3 (− 4.4, − 0.1)	0.041	− 217.5 (− 405.9, − 29.0)	0.025	− 3.3 (− 24.0, 17.4)	0.749
	MCS	− 1.5 (− 3.9, 0.9)	0.216	− 113.4 (− 327.4, 100.7)	0.292	3.6 (− 17.0, 24.1)	0.728
**Z-LTI**							
Z-LTI < 0 (n = 128)	KDCS	− 1.5 (− 4.5, 1.6)	0.341	13.8 (− 192.9, 220.5)	0.895	2.8 (− 24.1, 29.7)	0.837
	PCS	− 2.2 (− 3.9, − 0.5)	0.01	− 145.9 (− 259.6, − 32.2)	0.013	− 4.9 (− 20.6, 10.9)	0.54
	MCS	− 2.0 (− 3.9, − 0.1)	0.04	− 82.0 (− 204.0, 43.8)	0.206	− 7.9 (− 24.7, 8.8)	0.348
Z-LTI ≥ 0 (n = 69)	KDCS	− 1.4 (− 4.5, 1.8)	0.111	− 188.9 (− 4236.1, 48.2)	0.37	− 26.4 (− 65.4,12.5)	0.178
	PCS	− 1.3 (− 3.2, 0.6)	0.165	− 152.5 (− 291.3, − 13.8)	0.032	− 31.7 (− 54.2, − 9.2)	0.007
	MCS	− 0.4 (− 2.5, 1.6)	0.68	− 97.0 (− 250.4, 56.4)	0.21	− 16.4 (− 41.4, 8.6)	0.193

*HRQOL* health related quality of life, *ECW* extracellular water, *ICW* intracellular water, *Z-FTI* Z-score of fat tissue index, *Z-LTI* Z-score of lean tissue index, *CI* confidence interval, *KDCS* kidney disease component summary, *PCS* physical component summary, *MCS* mental component summary.

## Discussion

The present study demonstrated that an increase in hydration and fat tissue indices was negatively related to HRQOL scores in PD patients. These associations were significant after multivariable adjustment and were consistent throughout subgroup analysis stratified by sex, age, and daily urine. These findings indicate that interventions to improve hydration and fat tissue may potentially improve HRQOL in PD patients.

HRQOL is an important aspect of patient health status and should be considered when monitoring patients with chronic illness^20^. In particular, kidney diseases have negatively impacted the HRQOL of ESKD patients mainly due to the accompanying impairment or the imposed limitations in almost all aspects of their lives. Poor HRQOL is associated with increased morbidity and mortality^6^; thus, enhancing HRQOL is a priority in the area of kidney disease research^21,22^. There have been several studies on HRQOL and its implications among chronic kidney disease^23^, hemodialysis^6^, and transplanted patients^24–26^, but fewer studies have focused on PD patients^27^. In particular, to date, no study has reported the association of body composition and HRQOL in PD patients.

The body composition of PD patients differs from that of the general population^28^. Protein energy wasting and overhydration, which are common in PD patients, may affect changes in body composition. High glucose in dialysis fluid and a loss of proteins into the dialysate may contribute to a progressive decrease in skeletal muscle mass and increase in adipose tissue^29,30^. Although adverse changes in body composition are associated with morbidity and mortality in PD patients, there is no consensus on the relationship between body composition and HRQOL in PD patients. Moreover, PD patients with diabetes mellitus are known to have a high burden of both systemic and intraperitoneal inflammation^31^. In case of PD patients with residual renal function with high proteinuria and inflammatory status, serum albumin would be hard to reflect the nutritional status itself^32^. With an easy and noninvasive device to measure body composition, we tried to assess the association between body composition and HRQOL in PD patients.

In this study, we showed that R∆TH and ECW/ICW had significant negative associations with PCS after adjustments for multiple variables. The negative association between fluid overload and PCS showed robust relationships in multiple subgroup analyses stratified by sex, age, and dialysis urine. This result was consistent with a previous report^27^. As PCS is closely related with physical function, several reasons can be postulated. First, fluid overload in PD is not only related with cardiovascular burden but also non-cardiovascular risk factors such as malnutrition and inflammation^33,34^. Second, target weight may not always be reached due to an increase in intradialytic symptoms in fluid overload status, and fluid overloaded patients require more effort to adjust to their target weight^35^.

We also showed that FTI had a negative association with PCS after adjustments for multiple variables. PD patients are more susceptible to central obesity due to exposure to glucose-containing dialysate fluid^36^. However, in this analysis, when comparing subjects with Z-FTI < 0 and those with Z-FTI ≥ 0, the glucose exposure load was not significantly different between both groups (Supplement Table S2). The mechanism of negative association between PCS and Z-FTI has not been completely clarified, although several hypotheses could be inferred by considering the characteristics of the two groups classified as FTI (Supplement Table S2). First, patients with obese or high fat tissue secrete numerous proinflammatory cytokines, including C-reactive protein (CRP), tumor necrosis factor-α, and interleukin (IL)-6. Our study also showed that the lower Z-FTI group showed statistically significantly higher CRP than the higher Z-FTI group. These cytokines modulate lipid and carbohydrate metabolism and orchestrate the inflammatory pathway^37^. With this mechanism, a previous study showed that elevated CRP and IL-6 levels predict 2-year mortality, cardiovascular events, and technique survival in PD patients^30,38^. Second, a decline in nutritional status appears to have an impact on the physical domain of HRQOL in higher FTI groups. In our study, groups with Z-FTI ≥ 0 showed statistically significantly lower scores in SGA. Lower SGA has a vulnerability that is characterized by reduced functional reserve and a higher susceptibility to adverse health outcomes^39^. On the other hand, sarcopenic obesity may develop, where muscles decrease and fat increases in PD patients. In our subgroup analysis, it was confirmed that FTI and PCS had a significant negative association in subjects with higher Z-LTI, but not in those with lower Z-LTI. This association shows that the increased fat tissue itself, independent from the decrease in lean tissue mass, had a negative effect on PCS.

In contrast to the relationship between FTI and PCS, LTI did not show any relationship with HRQOL. This finding was different from a previous study reporting that a reduction of lean body mass was associated with an increased mortality^40^. Even though LTI did not show a significant association with HRQOL in our study, it should be interpreted with caution. Progressive loss of muscle mass and strength has frequently been observed in ESKD patients. Although this study did not prove any relationship between LTI and HRQOL, it is well known that LTI is an independent predictor of survival in PD^41^. From a long-term perspective, it is warranted to monitor the overall body composition of PD patients.

There was no association between KDCS and any of the body composition parameters in our study. Because the KDCS combines information from a heterogeneous set of scales^42^, each component has a different meaning to each of the body components. Even though MCS was weakly related with ECW/ICW and FTI, there was no linear trend between any of the body composition parameters and MCS. This finding was different from a previous study that found that severity of nutritional markers was related not only with PCS, but also with KDCS and MCS^43^. However, the nutritional status in the above study was evaluated based on a quantitative version of 24-h dietary recall and BMI, which have limitations in the accurate assessment of the body composition.

Our study had several limitations. First, this study was an observational study, and there were inherent limitations such as hidden confounding factors. For example, anemic conditions could affect the body composition and nutrition^44^. In our study, 79.2% of subjects were treated with erythropoiesis stimulating agents (ESA), and there was no statistically significant difference in the hemoglobin values in the group using ESA and the group not using ESA (11.0 ± 1.3 g/dL vs 10.7 ± 1.5 g/dL, p = 0.431). There are a variety of confounding factors that can affect the HRQOL in PD patients, and anemia in particular can affect the body composition and HRQOL. However, due to the cross-sectional observational study design, there is a possibility of hidden confounders. Second, a causal relationship could not be inferred due to the cross-sectional nature of the study. For example, we could not directly confirm whether the patient's satisfaction would change if the patient's body composition changes due to the influence of therapeutic effects. Even though we performed a linear regression analysis by adding the usage of RAAS blockade as an adjustment variable to model 5, there were inevitable drawbacks in that the causal relationship could not be confirmed. Further longitudinal or interventional studies are needed to better understand and determine the existence of a causal relationship. Third, the sample size of about 200 subjects may be insufficient to verify robust statistical significance. Nevertheless, this is the first study from PD patients evaluating the association between body composition and HRQOL. Moreover, we analyzed the body composition with Z-FTI and Z-LTI from Korean PD patients, using reference values from the Asian general population rather than the absolute values of FTI or LTI itself. This allowed us to investigate more accurately the association between body composition and HRQOL.

Based on the results of this study, it could be suggested that the implementation of strategies for the prevention and management of overhydration and obesity in PD patients could improve quality of life. Multidisciplinary strategies such as nutritional intervention, physical training, and psychological support for the HRQOL would be needed. Lifestyle and therapeutic adherence of PD patients who are overhydrated or obese could be considered in further investigations.

## Materials and methods

### Patients

This study is a cross-sectional and observational study to analyze the association between the body composition and HRQOL of PD patients enrolled in the KNOW-PD cohort. The KNOW-PD cohort is a single-center prospective cohort aimed at determining the prognosis of PD patients for which monitoring is planned for up to ten years beginning in 2015. In KNOW-PD cohort, patients (age ≥ 18 years) on maintenance PD were enrolled. Exclusion criteria were acute kidney disease, plans to undergo kidney transplant or transition to hemodialysis within 6 months, and pregnancy. Patients registered in the KNOW-PD cohort responded to HRQOL questionnaires at study entry and at 2, 4, and 8 years. The study subjects also underwent body composition measurement annually.

Among 363 patients who were enrolled KNOW-PD cohort, we excluded the subjects who were hybrid dialysis modalities (hemodialysis and PD) (n = 4), unable to undergo BIS because of defibrillators, artificial joints, pins, or limb amputations (n = 28), and those with the different measurement date for BIS and HRQOL (n = 134). Finally, a total of 197 PD patients who were eligible for data collection for both body composition and HRQOL were included.

### Measurement of body composition

Body composition was assessed using the BIS device (Body Composition Monitor [BCM]; Fresenius Medical Care, Bad Homburg, Germany). This device measures resistance, reactance, and phase angle from frequencies oscillating between 5 kHz and 1000 kHz^45^. The BCM was first validated against a gold standard to determine total body water (TBW), ECW, and ICW from the measured impedance data following the model of Moissl et al*.*^46,47^. The measurement was obtained by placing electrodes on one hand and one foot in a BCM device and entering current height and weight data into the device. Absolute ∆tissue hydration (A∆TH) represents the difference between the actual amount of ECW in the tissue as detected by the BCM and the amount of water present in tissue as predicted by physiological models under normal physiological conditions^48^. A∆TH is further normalized to ECW and expressed as a ratio called relative ∆tissue hydration (R∆TH = A∆TH/ECW). As a R∆TH ratio greater than 15% is related to mortality^49^, this cut-off was used to define “severe fluid overload”^45,50^. The BCM also provides the values of FTI, calculated as fat tissue mass/height^2^, and LTI, calculated as lean tissue mass/height^2^, by estimating the values of ICW, ECW, and TBW. FTI and LTI could be compared to the reference population, which is below the 10th percentile of about 2000 healthy adult subjects aged 18–75 years. We also expressed FTI and LTI as a Z-score, which was calculated by correcting age and sex for a population of 1294 Asian adults.

### Health-related quality of life measurement

HRQOL was evaluated using the Kidney Disease Quality of Life Short Form (KDQOL-SF) version 1.3^51^, which has been validated in many previous studies^6,24–27,52^. Patient responses to the KDQOL-SF were used to determine the scores of the kidney disease component summary (KDCS), physical component summary (PCS), and mental component summary (MCS). The questions used for the KDCS take into account particular kidney-related concerns of individuals with kidney diseases^53^. The scale for the KDCS was derived using 11 subscales: (1) symptoms/problems, (2) effects of kidney disease, (3) burden of kidney disease, (4) work status, (5) cognitive function, (6) quality of social interaction, (7) sexual function, (8) sleep, (9) social support, (10) dialysis staff encouragement, and (11) patient satisfaction. The scales for PCS and MCS were derived from eight different subscales, originally developed for the Short-Form Health Survey (SF-36)^54^. PCS was derived using 4 subscales: (1) physical functioning, (2) role-physical, (3) bodily pain, and (4) general health. MCS was derived using 4 subscales: (1) emotional well-being, (2) role-emotional, (3) social function, and (4) energy/fatigue. In all scales, a score from 0 to 100 was calculated, and a higher score indicates better quality of life.

### Data collection and ethical aspects

Baseline data and laboratory parameters were collected from the patient’s medical records. Dialysis adequacy with KT/Vurea per week, creatinine clearance, 24-h urine volume, and peritoneal membrane characteristics based on the results of the peritoneal equilibration test were collected. Information on glucose exposure load and usage of icodextrin was also collected. The Subjective Global Assessment (SGA), which is a tool that assesses nutritional status using medical history and a physical examination, was also administered^55^. In general, patients with SGA scores of 6–7, 3–5, and 1–2 are considered well nourished, mildly to moderately malnourished, and severely malnourished, respectively^56^. The Davies score, which is one of the comorbidity indexes, was recorded according to the protocol^57^. Left ventricular mass index were collected within 1 year from the study period from the echocardiography. The Institutional Review Board of Seoul National University Hospital (Institutional Review Board *No*. C-1506-097-681) approved the study. This study was conducted in accordance with the Declaration of Helsinki. All patients gave their informed consent to the study protocol before data collection and BIS measurement.

### Statistical analysis

Continuous variables were expressed as mean ± standard deviation or median and interquartile range according to normal or non-normal distribution. The normality of the distribution was assessed using the Kolmogorov–Smirnov test. Categorical variables were expressed as percentage of the total. Differences between groups stratified by body composition were determined using Student’s t-test for variables with normal distribution or Mann–Whitney test for variables with non-normal distribution. Spearman’s correlation analysis was used to determine the correlation between the parameter of body composition and the HRQOL scores. Linear regression testing was used to determine the association between body composition parameters and the HRQOL scores. Multivariate models included the significantly associated parameters according to their weight on univariate testing and clinically relevant parameters. A *p* value of < 0.05 was considered to indicate statistical significance, and statistical analysis was performed using SPSS software ver. 26.0 (IBM, Armonk, NY, USA).

## Supplementary Information

Supplementary Tables.

## Data Availability

The data that support the findings of this study are available from the corresponding author upon reasonable request.
