# Supplementary material for: Associations among body composition parameters and quality of life in peritoneal dialysis patients
Source: Sci Rep. 2022 Nov 10;12:19192. doi: 10.1038/s41598-022-19715-2 (PMC9649675; doi:10.1038/s41598-022-19715-2)
Supplement: Supplementary file 1 — Supplementary Tables. [file 41598_2022_19715_MOESM1_ESM.docx]

| **Supplement Table S1. Subscale of HRQOL classified according to body composition** | | | | | | | | | | | | |
| --- | --- | --- | --- | --- | --- | --- | --- | --- | --- | --- | --- | --- |
|  | **Relative △Tissue Hydration** | | | **ECW/ICW** | | | **Z-FTI** | | | **Z-LTI** | | |
|  | R△TH ≤ 15%  (n=118) | R△TH> 15%  (n=79) | *p* | ECW/ICW <1  (n=144) | ECW/ICW ≥1  (n=53) | *P* | Z-FTI < 0  (n=121) | Z-FTI ≥ 0  (n=76) | *p* | Z-LTI < 0  (n=128) | Z-LTI ≥ 0  (n=69) | *p* |
| Symptom/problems | 81.3  (62.5,89.6) | 81.3  (70.8,85.4) | 0.581 | 81.3  (64.6,87.5) | 81.3  (68.8,87.5) | 0.95 | 81.3  (64.6,87.5) | 81.3  (67.2,87.5) | 0.957 | 81.3  (68.8,87.5) | 73.8±14.4 | 0.048 |
| Effects of kidney disease | 75.0  (58.8,86.2) | 68.8  (50.0,81.3) | 0.02 | 71.9  (56.3,84.4) | 71.9  (48.4,85.0) | 0.911 | 69.1±19.9 | 71.9  (50.0,81.3) | 0.498 | 75.0  (57.7,84.4) | 64.9±20.7 | 0.062 |
| Burden of kidney disease | 40.6  (25.0,62.5) | 31.3  (18.8,50.0) | 0.007 | 37.5  (18.8,56.3) | 31.3  (18.8,50.0) | 0.368 | 37.5  (18.8,56.3) | 31.3  (18.8,50.0) | 0.207 | 37.5  (18.8,56.3) | 31.3  (18.8,56.3) | 0.953 |
| Work status | 50.0  (0.0,100.0) | 50.0  (0.0,50.0) | 0.423 | 50.0  (0.0,100.0) | 50.0  (0.0,50.0) | 0.385 | 50.0  (0.0,100.0) | 50.0  (0.0,50.0) | 0.306 | 50.0  (0.0,87.5) | 50.0  (0.0,100.0) | 0.282 |
| Cognitive function | 93.3  (85.0,100.0) | 93.3  (80.0,100.0) | 0.075 | 93.3  (80.0,100.0) | 93.3  (76.7,100.0) | 0.589 | 93.3  (80.0,100.0) | 93.3  (75.0,100.0) | 0.645 | 93.3  (86.7,100.0) | 93.3  (76.7,100.0) | 0.656 |
| Quality of social interaction | 66.7  (60.0,80.0) | 66.7  (60.0,80.0) | 0.976 | 66.7  (60.0,80.0) | 66.7(  60.0,80.0) | 0.999 | 66.7  (60.0,80.0) | 66.7  (53.3,86.7) | 0.841 | 66.7  (60.0,80.0) | 66.7  (60.0,80.0) | 0.772 |
| Sexual function | 0.0  (0.0,0.0) | 0.0  (0.0,50.0) | 0.144 | 0.0  (0.0,0.0) | 0.0  (0.0,75.0) | 0.073 | 0.0  (0.0,25.0) | 0.0  (0.0,50.0) | 0.314 | 0.0  (0.0,18.8) | 0.0  (0.0,50.0) | 0.222 |
| Sleep | 63.3±18.1 | 60.5±21.9 | 0.333 | 62.9±19.0 | 60.3±21.7 | 0.426 | 62.3±18.2 | 62.0±22.1 | 0.94 | 63.2±20.6 | 60.3±18.0 | 0.318 |
| Social support | 66.7  (66.7,87.5) | 66.7  (50.0,100.0) | 0.666 | 66.7  (50.0,83.3) | 66.7  (66.7,100.0) | 0.439 | 66.7  (66.7,91.7) | 66.7  (50.0,100.0) | 0.865 | 66.7  (66.7,100.0) | 66.7  (50.0,83.3) | 0.442 |
| Dialysis staff encouragement | 100.0  (75.0,100.0) | 100.0  (75.0,100.0) | 0.859 | 100.0  (75.0,100.0) | 100.0  (75.0,100.0) | 0.461 | 100.0  (75.0,100.0) | 100.0  (87.5,100.0) | 0.015 | 100.0  (75.0,100.0) | 100.0  (75.0,100.0) | 0.285 |
| Patient satisfaction | 83.3  (66.7,100.0) | 66.7  (66.7,100.0) | 0.09 | 83.3  (66.7,100.0) | 66.7  (66.7,91.7) | 0.353 | 83.3  (66.7,100.0) | 83.3  (66.7,100.0) | 0.266 | 83.3  (66.7,100.0) | 83.3  (66.7,83.3) | 0.982 |
| Physical functioning | 90.0  (80.0,95.0) | 85.0  (80.0,95.0) | 0.026 | 90.0  (80.0,95.0) | 85.0  (65.0,95.0) | 0.02 | 90.0  (80.0,95.0) | 85.0  (76.3,95.0) | 0.228 | 90.0  (80.0,95.0) | 85.0  (80.0,95.0) | 0.655 |
| Role-physical | 25.5  (0.0,100.0) | 25.0  (0.0,75.0) | 0.059 | 25.5  (0.0,100.0) | 0.0  (0.0,75.0) | 0.041 | 25.5  (0.0,100.0) | 25.0  (0.0,75.0) | 0.062 | 25.0  (0.0,100.0) | 25.0  (0.0,75.0) | 0.166 |
| Bodily pain | 70.0  (45.0,90.0) | 67.5  (45.0,90.0) | 0.125 | 70.0  (47.5,90.0) | 67.5  (35.0,85.0) | 0.054 | 70.0  (45.0,90.0) | 67.5  (45.0,90.0) | 0.379 | 67.5  (45.0,90.0) | 70.0  (45.0,90.0) | 0.705 |
| General health | 40.0  (25.0,50.0) | 30.0  (20.0,45.0) | 0.004 | 35.0  (21.3,50.0) | 30.0  (20.0,50.0) | 0.124 | 35.0  (20.0,50.0) | 30.0  (20.0,50.0) | 0.179 | 34.2  (20.0,50.0) | 36.3±18.0 | 0.898 |
| Emotional well-being | 62.1±18.6 | 60.0  (52.0,68.0) | 0.212 | 60.0(48.0,75.0) | 62.7±16.3 | 0.784 | 64.0  (52.0,76.0) | 58.9±18.8 | 0.146 | 60.0  (49.0,76.0) | 60.1±19.5 | 0.983 |
| Role-emotional | 100.0  (33.3,100.0) | 66.7  (0.0,100.0) | 0.09 | 66.7  (0.0,100.0) | 66.7  (0.0,100.0) | 0.13 | 100.0  (0.0,100.0) | 66.7  (0.0,100.0) | 0.172 | 100.0  (0.0,100.0) | 66.7  (0.0,100.0) | 0.22 |
| Social function | 62.5  (50.0,75.0) | 50.0  (37.5,75.0) | 0.056 | 62.5  (40.6,75.0) | 62.0±24.8 | 0.675 | 62.5  (50.0,75.0) | 62.5  (37.5,75.0) | 0.268 | 62.5  (37.5,75.0) | 62.5  (50.0,75.0) | 0.228 |
| Energy/fatigue | 50.0  (30.0,60.0) | 45.0  (30.0,55.0) | 0.186 | 45.0  (30.0,55.0) | 50.0  (30.0,57.5) | 0.834 | 50.0  (32.5,57.5) | 45.0  (30.0,55.0) | 0.172 | 45.0  (30.0,55.0) | 50.0  (30.0,60.0) | 0.607 |
| Abbreviation: HRQOL, health related quality of life; R△TH, Relative △tissue hydration; ECW, extracellular water; ICW, intracellular water; Z-FTI, Z-score of fat tissue index; Z-LTI, Z-score of lean tissue index; KDCS, kidney disease component summary; PCS, physical component summary; MCS, mental component summary | | | | | | | | | | | | |

| **Supplement Table S2. Baseline demographic and biochemical details of the study subjects classified into Z-FTI** | | | |
| --- | --- | --- | --- |
| Characteristics | Z-FTI < 0  (n=121) | Z-FTI ≥ 0  (n=76) | *p* |
| Male | 62 (51.2%) | 46 (60.5%) | 0.202 |
| Age (years) | 45.0±13.0 | 47.1±14.3 | 0.288 |
| Height (cm) | 162.8±8.5 | 165.6±8.6 | 0.025 |
| Weight (kg) | 56.0±10.4 | 71.6±11.0 | <0.001 |
| Blood pressure |  |  |  |
| Systolic (mmHg) | 138.4±18.7 | 141.6±21.4 | 0.281 |
| Diastolic (mmHg) | 84.9±13.1 | 82.5±13.7 | 0.222 |
| Dialysis duration (month) | 39 (1.5, 77) | 21.5 (0, 53.8) | 0.01 |
| Peritoneal dialysis modality |  |  |  |
| Continuous ambulatory peritoneal dialysis | 57 (47.1%) | 35 (46.1%) | 0.727 |
| Continuous cycling peritoneal dialysis | 35 (28.9%) | 18 (23.7%) |  |
| Intermittent peritoneal dialysis | 23 (19.0%) | 19 (25.0%) |  |
| Night intermittent peritoneal dialysis | 6 (5.0%) | 4 (5.3%) |  |
| Kt/V |  |  |  |
| Peritoneal | 1.65 (1.3, 1.8) | 1.4±0.5 | <0.001 |
| Renal | 0.2 (0,0.8) | 0.5 (0, 1.0) | 0.175 |
| Total | 1.9 (1.7, 2.3) | 2.0±0.5 | 0.27 |
| Creatinine clearance (ml/min/1.73m^2^) |  |  |  |
| Peritoneal | 42.2 (31.8, 48.8) | 37.9 (24.8, 47.7) | 0.195 |
| Renal | 17.0 (0, 43.5) | 25.3 (0, 67.6) | 0.071 |
| Total | 54.1 (46.4, 72.6) | 57.3 (43.3, 84.6) | 0.819 |
| 24-hr urine volume (ml/day) | 300(0,1000) | 450 (0, 1137.5) | 0.226 |
| Glucose exposure load (g/day) | 89.4 (69.9, 114.7) | 88.3 (58.9, 112.5) | 0.372 |
| History of icodextrin bag usage | 57 (47.1%) | 33 (56.6%) | 0.196 |
| Type of membrane transport |  |  |  |
| High | 28 (25.9%) | 29 (33.8%) | 0.086 |
| High Average | 42 (38.9%) | 24 (33.8%) |  |
| Low Average | 26 (24.1%) | 16 (22.5%) |  |
| Low | 12 (11.1%) | 2 (2.8%) |  |
| Missing | 13 | 5 |  |
| Subjective global assessment |  |  |  |
| 4-5 | 2 (1.7%) | 6 (8.1%) | 0.026 |
| 6 | 21 (18.1%) | 20 (27.0%) |  |
| 7 | 93 (80.2%) | 48 (64.9%) |  |
| Missing | 5 | 2 |  |
| Diabetes mellitus | 18 (14.9%) | 32 (42.1%) | <0.001 |
| Davies score |  |  |  |
| 1 | 65 (53.7%) | 33 (43.4%) | 0.222 |
| 2-3 | 55 (45.5%) | 41 (45.7%) |  |
| ≥4 | 1 (0.8%) | 2 (2.6%) |  |
| Laboratory finding |  |  |  |
| Albumin(g/dL) | 3.8±0.5 | 3.7±0.4 | 0.447 |
| BUN (mg/dL) | 58.4±15.4 | 56.9±17.4 | 0.523 |
| C-reactive protein (mg/dL) | 0.08 (0.03, 0.23) | 0.12 (0.05, 0.33) | 0.032 |
| Hemoglobin (g/dL) | 10.7±1.5 | 10.8±1.5 | 0.654 |
| Sodium (mmol/L) | 138 (135, 140) | 138 (134, 141) | 0.826 |
| Potassium (mmol/L) | 4.5±0.7 | 4.5±0.8 | 0.936 |
| Cholesterol (mg/dL) | 170.2±37.8 | 143.5 (119.8, 188.8) | 0.027 |
| Bioimpedance measurements |  |  |  |
| Total body water (L) | 33.0±7.6 | 36.3±7.4 | 0.003 |
| Extracellular water (L) | 15.2±3.8 | 17.9±3.8 | <0.001 |
| Intracellular water (L) | 17.7±4.3 | 18.3±4.1 | 0.356 |
| Extracellular water/ Intracellular water | 0.9 (0.8, 0.9) | 1.0±0.1 | <0.001 |
| Relative △tissue hydration (R△TH, %) | 12.2±9.9 | 15.3±10.4 | 0.042 |
| Absolute △tissue hydration (A△TH, L) | 1.6(0.8, 2.8) | 3.0±2.3 | 0.004 |
| Fat tissue index (kg/m^2^) | 5.5±2.3 | 10.9 (8.9, 13.0) | <0.001 |
| Z-score of fat tissue index | -0.7 (-1.3, -0.4) | 0.7 (0.3, 1.3) | <0.001 |
| Lean tissue index (kg/m^2^) | 14.7±3.0 | 13.7±2.8 | 0.017 |
| Z-score of lean tissue index | -0.2±1.1 | -0.9±1.2 | <0.001 |
| Continuous variables are expressed as mean ± standard deviations (normal distribution) or as median and interquartile (non-normal distribution). Categorical variables are expressed as number, (%) | | | |
